# European meeting in the field of history of medicine: Biennial Conference of the European Association for the History of Medicine and Health

**Published:** 2017

**Authors:** VL Purcarea

**Affiliations:** * "Carol Davila" University of Medicine and Pharmacy, Bucharest, Romania

**Biennial Conference of the European Association for the History of Medicine and Health** – *The Body Politic: States in the History of Medicine and Health*, an extremely important European meeting in the field of history of medicine, whose main organizer was the European Association for the History of Medicine and Health, took place in the amphitheatre of “Carol Davila” University of Medicine and Pharmacy, Bucharest, during August 30 and September 02, 2017.

Hosted for the first time by a East European country, the conference enjoyed the participation of some remarkable personalities in the academic world such as doctors, historians, anthropologists, demographers, and occasioned an interesting and useful exchange of opinions, mainly regarding the role of politics in the development of medicine in history, benefitting from the participation of some prestigious publishing houses and raising the interest of many participants.

**Fig. 1:**
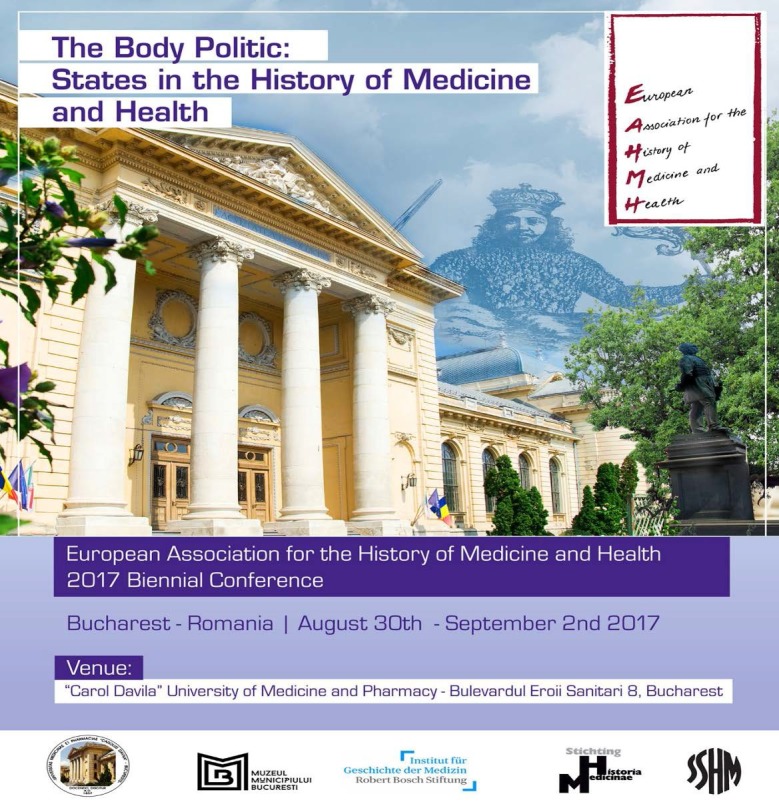
Poster of the conference hosted by the European Association for the History of Medicine and Health

Prof. Dr. Octavian Buda, President of the European Association for the History of Medicine and Health, EAHMH, and the Head of Discipline of History of Medicine in the Preclinical 3 Department, “Complimentary Sciences”, affirmed: “I hope this conference will have positive outcomes, so that we will be able to raise the interest of the researchers in the Occident regarding the study of history of medicine of these complex fields in the Eastern Europe, such as Romania, with the starting of some academic collaboration projects between the Western and Eastern Europe”.

**Fig. 2:**
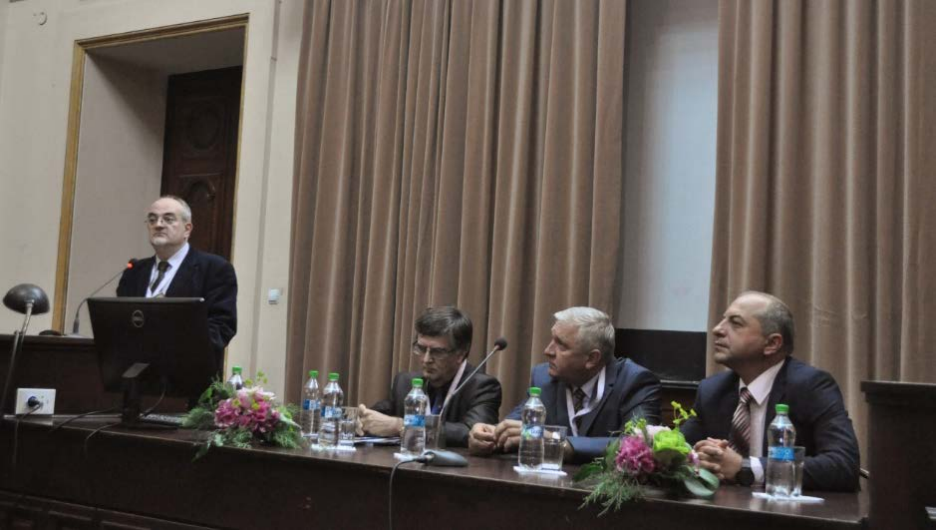
Presidium of the conference. Speaker: Prof. Dr. Octavian Buda

Prof. Dr. Mircea Beuran, President of the Senate of “Carol Davila” University of Medicine and Pharmacy, Bucharest performed the welcoming speech of the conference. Other speakers were Prof. Dr. Catalin Cirstoiu, Dean of “Carol Davila” University of Medicine and Pharmacy, Bucharest; Prof. Dr. Mircea Dumitru, Rector of the University of Bucharest and Prof. Dr. Octavian Buda, President of EAHMH.

The conference took place under the patronage of two important academic institutions: Academy of Medical Sciences and Academy of Scientists, and reunited over 150 participants from 22 countries, also enjoying the presence of four keynote speakers.

**Fig. 3:**
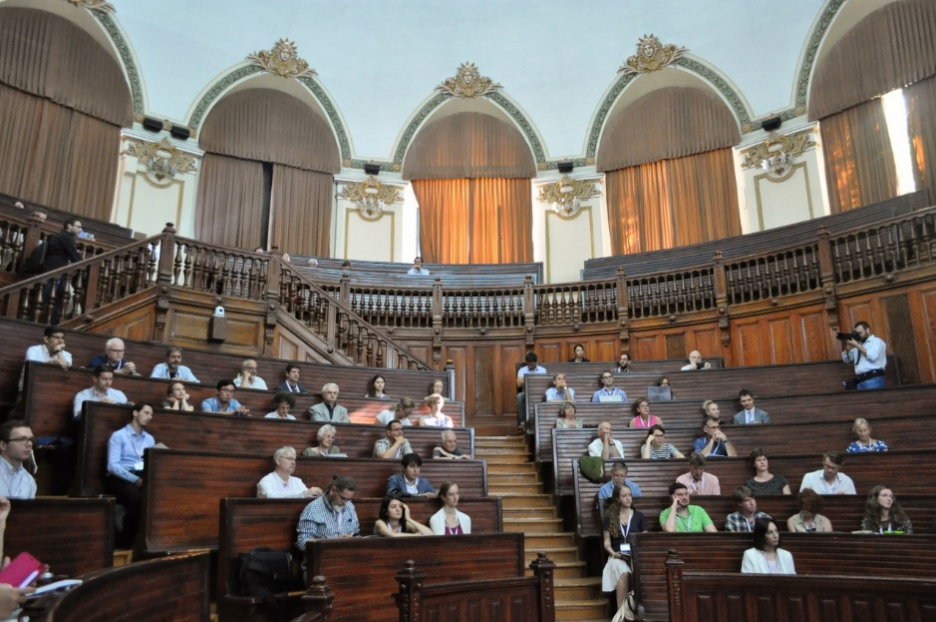
Participants in the event

Among the most important personalities who activate in the fields of human and medical sciences and took part in the event were Prof. Dr. Roberta Bivins - University of Warwick, England; Prof. Dr. Harry Oosterhuis - University of Maastricht, Netherlands; Prof. Dr. Constantin Goschler – University of Bochum, Germany and of course, Prof. Dr. Mircea Dumitru, Rector of the University of Bucharest.

2017 EAHMH Book Award Prize was awarded for the best book published in the field of history of medicine and went to Cristian Berco for his book “From Body to Community. Venereal Disease and Society in Baroque Spain”, published by University of Toronto Press in 2016 and Pieter van Foreest Student Prize, which is offered to students and PhD students for the best paper presented in the conference, prize that was awarded to Kinga Jeczminska for the paper “Political Factors in Abandoning Lobotomy in Poland in the 1950s”.

**Executive Editor** **Professor Eng. Victor Lorin Purcarea, PhD.**

